# Three Metachronous Osteosarcomas within 22 Years without Pulmonary Metastases: A Case Report and Review of the Literature

**DOI:** 10.1155/2013/197287

**Published:** 2013-12-25

**Authors:** Ulrike Michaela Pirker-Frühauf, Jörg Friesenbichler, Katharina Rabitsch, Bernadette Liegl-Atzwanger, Thomas Bauernhofer, Reinhard Windhager, Andreas Leithner

**Affiliations:** ^1^Department of Orthopaedic Surgery, Medical University of Graz, Auenbruggerplatz 5-7, 8036 Graz, Austria; ^2^Department of Pathology, Medical University of Graz, Auenbruggerplatz 25, 8036 Graz, Austria; ^3^Division of Oncology, Department of Internal Medicine, Medical University of Graz, Auenbruggerplatz 15, 8036 Graz, Austria; ^4^Department of Orthopaedic Surgery, Medical University of Vienna, Währinger Gürtel 18-20, 1090 Wien, Austria

## Abstract

*Introduction*. We present the extremely rare case of a patient with three metachronous osteosarcomas within 22 years without evident pulmonary manifestation of disease 30 years after first diagnosis. *Case Presentation*. In 1983, a high-grade osteosarcoma of the left distal femur was diagnosed in an 18-year-old Caucasian male. He received rotationplasty accompanied by pre- and postoperative chemotherapy. Ten years later, an osteoblastic osteosarcoma occurred in TH12. En bloc resection and pre- and postoperative chemotherapy followed. In 2005, the patient developed another high-grade osteosarcoma in his right distal femur. Treatment included a wide resection and reconstruction with a tumour endoprosthesis as well as (neo)adjuvant chemotherapy. After the third tumour occurrence, cytogenetic and molecular genetic examinations (p53, rb1) were performed, showing a normal genetic pattern. Screening for metastases never showed clinical evidence of extraskeletal tumour manifestation. *Discussion*. In patients presenting metachronous osteosarcoma, identification of their lesions clonality (second primary tumour or metastases) could lead to a better understanding of tumour development and help to filter patients who need extended long-term followup due to a higher risk of late occurring sarcoma recurrence.

## 1. Introduction

Osteosarcoma occurs with an incidence of four to five cases per million inhabitants per year, representing the most common nonhaematopoietic primary malignant bone tumour [1]. It is developing most frequently in the second decade of life with approximately 60% of patients being younger than 25 years [1]. About 80% of those patients already present subclinical micrometastases at the time of primary tumour diagnosis [2]. Therefore, pre- and postoperative chemotherapeutic treatment in addition to a wide resection, and in exceptional cases of inappropriate resectability also local radiotherapy, has been documented as a successful therapeutic approach [3, 4]. This interdisciplinary therapy concept contributed to an increase of the 5-year survival rate of osteosarcoma patients from 15% to 50–70% [5].

Nevertheless, some patients still experience recurrences, and of those, less than one quarter become long-term survivors [3]. The highest probability of tumour recurrence, independent of the localisation, has been reported during the first three years after initial osteosarcoma diagnosis [4]. Generally, osteosarcoma is thought to spread on a haematogenous pathway primarily to the lungs, with the skeleton presenting the second most common organ of metastatic manifestation [2]. In some rare cases, visceral or cerebral metastases were reported [6]. Osteosarcomatous lesions, which occur distant from the primary site of osteosarcoma affection after initial treatment without pulmonary manifestation, are classified as metachronous osteosarcoma with reported incidence rates varying from less than 1% [6] to 10% [7] among all osteosarcoma cases [2, 3, 7]. Reports on two or more relapses, as presented in our case, are sparse [8]. Therapy of those lesions is difficult due to the necessity of individual decisions, depending on the already given primary treatment. Patients with late recurrences are reported to have a more favourable outcome if treated according to the principles of primary osteosarcoma [4].

The pathogeneses of metachronous osteosarcoma are still unclear. They are discussed either as metastases of the primary tumour to the bone or second primary lesions. Histopathology does not offer sufficient methods of differentiation yet [3]. Theories on its development, without involvement of the lungs, are focusing on a bone-to-bone-spread via venous plexus, intraosseous embolisation, or lymphatic spread [2]. A predisposition to primary osteosarcoma development has been observed in patients presenting genetic abnormalities of the p53 tumour suppressor pathway (e.g., Li-Fraumeni-like syndrome) or retinoblastoma gene germline mutations. Nevertheless, those alterations are discussed contradictorily in studies focusing on metachronous lesions [9–11].

Herein, we present the extremely rare case of a patient with three osteosarcomas within 22 years in different sites of the skeletal system without any other manifestation of disease 30 years after first diagnosis.

## 2. Case Presentation

In 1983, a high-grade osteosarcoma of the left distal femur was diagnosed by biopsy in an 18-year-old Caucasian male at the University Hospital Vienna. The patient did not show evidence of pulmonary metastases. He received preoperative chemotherapy according to the Rosen T10 protocol, based on high-dose Methotrexate monotherapy, showing a regression grade of five (>50% vital tumour tissue) on a six-part scale system according to Salzer Kuntschik et al. (1 = no viable tumour cells, 6 = no response to chemotherapy) [12]. Rotationplasty was performed in addition to adjuvant chemotherapy based on eight cycles of Cisplatin and Adriamycin.

Ten years later, in 1993, an osteoblastic osteosarcoma occurred in the twelfth thoracic vertebral body, again without any other sign of systemic manifestation of disease. After preoperative chemotherapy using Carboplatin/Etoposide, the patient received en bloc resection and dorsal stabilisation from TH12 to L3 at the same institution. Histologic workup revealed a regression grade of three according to the Salzer Kuntschik scale and postoperative adjuvant chemotherapy with Adriamycin for one cycle and Carboplatin/Etoposide for 4 cycles was administered.

**Figure 1:**
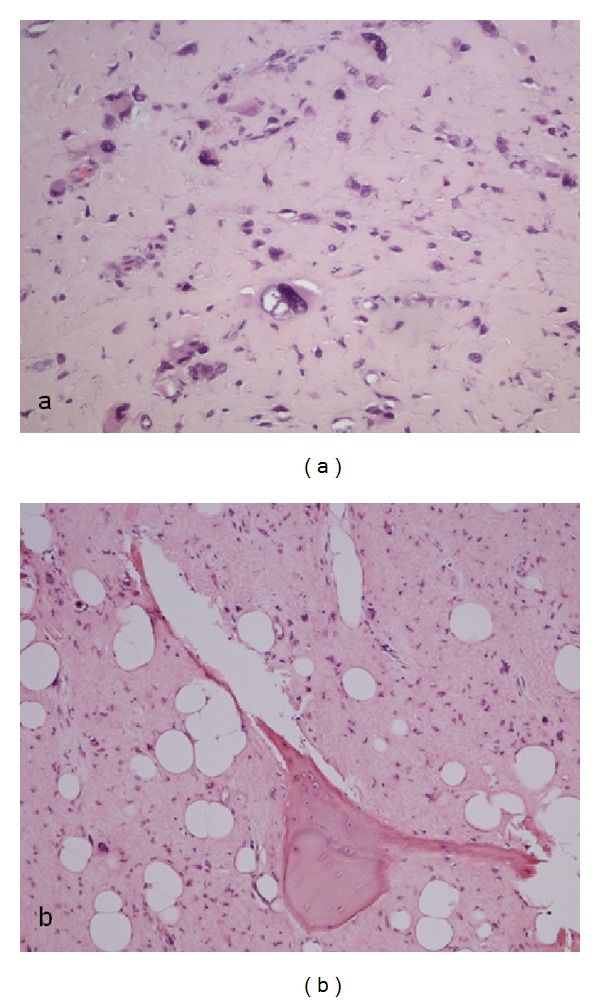
Histopathological pictures of the last high-grade osteosarcoma of the right distal femur in 2005 (a) before and (b) after chemotherapeutic application.

In 2005, 12 years after vertebral resection, the same patient showed swelling and pain in his right distal femur. The following biopsy at the University Hospital Graz revealed again a high-grade osteosarcoma (Figure 1(a)), and once more, screening showed no evidence of any extraskeletal tumour suspect lesions. The wide resection and tumour endoprosthetic reconstruction were preceded by neoadjuvant chemotherapy, based on Carboplatin/Etoposide, high-dose Methotrexate, and Hycamtin/Endoxan. A regression grade of 5 was achieved (Figure 1(b)), identical to the first osteosarcoma response. Postoperative a combination of Carboplatin/Etoposide, high-dose Methotrexate, and Ifosfamide/Etoposide was given.

**Figure 2:**
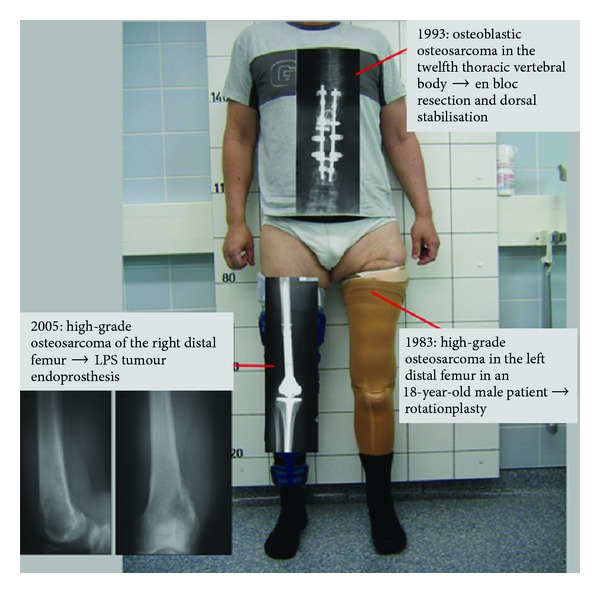
Overview about localisation, timely occurrence, and operative intervention of the three metachronous osteosarcomas as in our 48-year-old male patient 30 years after first osteosarcoma diagnosis.

Mobilisation was difficult due to the rotationplasty of 1983 on the left leg and the recently implanted tumour endoprosthesis on the right one. Two months after the last operation during the ongoing adjuvant chemotherapy, our patient fell and suffered from an open luxation of the recently operated knee. Revision surgery with change of prosthesis was necessary. After the revision procedure, no further complications occurred and the patient is now able to walk without braces or canes representing a good functional outcome (Figure 2). At the most recent followup, 8 years after the last relapse and 30 years after first diagnosis, the patient is still free of tumour recurrence.

After the second tumour relapse, we performed cytogenetic and molecular genetic examination after obtaining our patient's written informed consent. Based on the major genetic alterations mentioned in the literature as predisposing to osteosarcoma development, we were focusing on Li-Fraumeni-like syndrome (p53 gene mutation) and retinoblastoma syndrome (RB 1 gene mutation), but no mutations in the target genes could be found.

## 3. Discussion

Development of interdisciplinary osteosarcoma treatment has increased patients survival and the event free interval concerning tumour relapse. Nevertheless, patients still experience tumour recurrence. In this context, pulmonary metastases, with or without secondary bone lesions, are seen more frequently than cases of solitary osseous recurrences [3]. Reports on two or more relapses, as the one presented here, are sparse [8]. In those metachronous osteosarcoma cases, different tumour biology is suspected, but due to a lack of sufficient histopathological methods to evaluate clonality, clinicians cannot distinguish between real bone metastases and second primary osteosarcoma [3]. This information might be of high value concerning treatment decisions [3].

Generally, the prognosis of patients who relapse with bone lesions is worse than that of patients with pulmonary metastases. In a study cohort of 52 patients at the Rizzoli Institute, only patients with single, late appearing, and resectable bone malignancies presented a similar outcome compared to patients with secondary lesions to the lungs [7]. Aung et al. [4] described a 5-year postrelapse overall survival of 61% in 11 patients with late (>24 mo) metachronous tumours compared to 8% in 12 patients with early (≤24 mo) lesions. Franke et al. [3] reported a 5-year postrelapse overall survival of 54% in a cohort of 38 patients.

A possible interpretation concerning the poorer outcome in patients with early recurrence (<24 months), compared to those with late occurring tumours (>24 months), was given by Brandal et al. [2]. They stated that early lesions might present real metastases, whilst late lesions are more likely to be clonally independent of the primary tumour. In our patient, both relapses occurred more than 24 months after the primary malignancy which is, according to Brandal, more a hint that he developed three “real primary” osteosarcomas than bone metastases.

Besides worse life expectancy, Bacci et al. [7] found a higher rate of local recurrences in patients with bone metastases (36%), compared to those whose malignancies spread to the lungs (7%), among 52 patients with skeletal metastases and 371 patients with pulmonary lesions. The authors concluded that this might indicate different tumour biology of the primary osteosarcoma [7].

Rodriguez et al. [6] stated three possible pathophysiological pathways which might explain the limited metastatic capacity to the bone: first, a change of the primary osteosarcoma by chemotherapeutic treatment, second, a special genetic characteristic of the tumour itself, and, third, a genetic alteration in the patient.

Conditions predisposing to osteosarcoma development, including tumour suppressor pathway alterations of the p53 gene, finding clinical presentation in the Li-Fraumeni-like syndrome, and retinoblastoma gene 1 germline mutations, are well described in literature in the context of primary osteosarcoma development [9]. Nevertheless, this genetic influence is discussed contradictorily in metachronous osteosarcoma. Jaffe et al. [8] reported three cases of bilateral retinoblastoma and one with Li-Fraumeni-like syndrome in a cohort of 11 patients with solitary osseous osteosarcoma recurrences, whilst Franke et al. [3] presented only one case with bilateral retinoblastoma out of 38 patients. In our patient, a genetic component seems to be obvious, but neither genetic alterations coinciding with Li-Fraumeni-like syndrome (p53 tumour suppressor pathway) nor mutations of the retinoblastoma gene 1 could be found.

Another hypothesis concerning the differentiation of tumour metastases from second primary lesions was given by Franke et al. [3]. They assumed that primary lesions were mainly affecting the extremities, whilst their osseous relapses showed a predilection for axial and craniofacial sites. Taking this into consideration, the first tumour recurrence in our patient, which was localized in the spine, might have represented a metastatic lesion, as this was also hypothesized by our pathologist after careful histologic examination of the specimens of the left femur and the 12th vertebra [13]. The second relapse in the contralateral femur in 2005 might have been a second primary tumour.

On the other hand, the two tumours in the lower limbs were both responding to chemotherapy with a regression grade of five (>50% viable tumour cells) according to Salzer Kuntschik [12], whilst the lesion in the spine showed a much better response with a regression grade of three. This would more support a clonal relationship between the two malignancies in the extremities than that between the primary tumour and the vertebral relapse. Nevertheless, the grade of chemotherapeutic regression will not prove or disprove clonality in our case due to the diversity of the treatment protocols used for the single lesions. Further, metastases might show dissimilarity to the primary tumour and respond differently to chemotherapeutic application [2]. Reported cases of different levels of necrosis in recurrences might be explained by chemotherapeutic adjustment [2].

The treatment of metachronous osteosarcoma remains difficult. San-Julian et al. [14] suggested a curative attempt, including surgery and chemotherapy, in solitary lesions. We acknowledge this as our patient presents the potential benefit. Eight years after the last resection and 30 years after first osteosarcoma diagnosis, he is still free of recurrence and shows a good functional outcome.

## 4. Conclusion

The precise etiology of metachronous osteosarcoma still remains unclear. In our case, a genetic component is obvious, although we could not find an association with the so far known osteosarcoma related main gene mutations. Identification of those lesions' clonality (second primary tumours versus metastases) could lead to a better understanding of tumour development and help to filter patients who need extended long-term followup due to a higher risk of late occurring sarcoma recurrence.
